# SIRT1 mediates KU70 to maintain genomic stability in spermatogonial stem cells via the NHEJ repair pathway

**DOI:** 10.1038/s41419-026-08710-4

**Published:** 2026-04-07

**Authors:** Fang Zhou, Yanli Xiao, Qiaorui Yang, Wenhan Ju, Xin Xin, Qiong Wang, Yan Hong, Wei Le, Jinfu Zhang

**Affiliations:** 1 Department of Surgery, Shenzhen Luohu Women and Children’s Healthcare Hospital, Shenzhen Luohu Hospital Group, Shenzhen, China; 2 Department of Gynecology, Shenzhen Luohu Hospital of Traditional Chinese Medicine, Shenzhen Luohu Hospital Group, Shenzhen, China; 3 Department of Gynecology, Guanghua Hospital Affiliated to Shanghai University of Traditional Chinese Medicine, Shanghai, China; 4 Department of Traditional Chinese Medicine, Tongji Hospital, School of Medicine, Tongji University, Shanghai, China; 5 Department of Urology, Tongji Hospital, School of Medicine, Tongji University, Shanghai, China

**Keywords:** Stress signalling, Infertility

## Abstract

Male infertility is closely related to DNA double-strand breaks in spermatogonial stem cells (SSCs); however, the precise mechanism still remains to be fully elucidated. While SIRT1 is a key regulator of DNA damage response and cellular senescence in other contexts, its role in SSCs is still poorly understood. In this study, human testicular single-cell RNA sequencing datasets were reanalyzed to characterize SSC transcriptional programs in non-obstructive azoospermia (NOA) patients. Clinical validation was performed on testicular sections from obstructive azoospermia controls and NOA patients. X-ray irradiation and hydroxyurea-based DNA damage models were applied to interrogate SSC DNA damage responses in vivo and in vitro. Immunofluorescence, western blotting, co-immunoprecipitation, growth and survival assays, flow cytometry, a GFP-based NHEJ reporter, and acetylation analyses were used to define SIRT1-associated pathways. Single-cell analysis revealed an overall attenuation of NHEJ-related signatures and reduced SIRT1 expression in SSCs from NOA compared with controls. In clinical specimens, confocal immunofluorescence confirmed a reduced SSC pool and decreased SIRT1 and 53BP1 signals within PLZF-positive SSCs, while KU70 levels were not significantly changed. In experimental models, acute DNA damage induced a rapid SIRT1 response in SSCs. Functional assays showed that SIRT1 supports SSC homeostasis by promoting proliferative capacity and influencing apoptosis and survival under hydroxyurea-induced DNA damage. Mechanistically, SIRT1 co-localized and physically interacted with KU70, with enhanced association under genotoxic stress. NHEJ reporter assays showed reduced repair efficiency following *Sirt1* knockdown. Moreover, *Sirt1* overexpression may down-regulate KU70 acetylation, indicating a deacetylation-dependent mechanism in NHEJ regulation. Collectively, these findings identify SIRT1 as a stress-responsive regulator of SSC genome maintenance that functionally cooperates with KU70 to support NHEJ-associated repair and limit DNA damage-driven SSC loss. The SIRT1–KU70 axis represents a potential target to mitigate genotoxic injury–associated germline stem cell attrition and preserve male fertility.

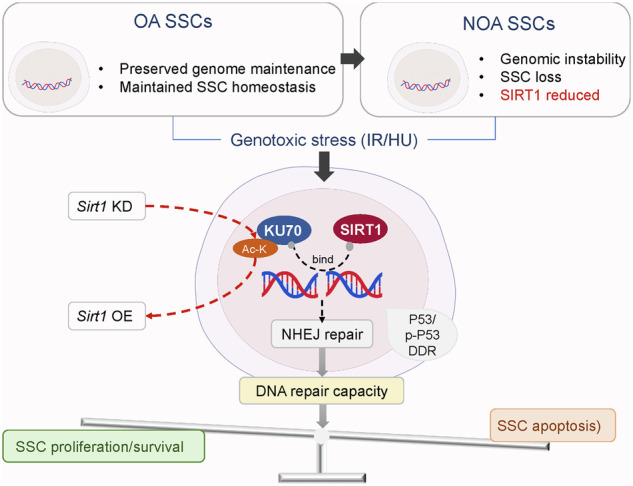

## Introduction

Male infertility represents a significant global health challenge, affecting approximately 7% of men and contributing to nearly half of all infertility cases, with a primary etiology in impaired spermatogenesis [1]. This dysfunction frequently manifests as severe conditions such as non-obstructive azoospermia (NOA) [2]. Spermatogonial stem cells (SSCs) are responsible for sustaining spermatogenesis throughout adulthood [3]; however, they exhibit high vulnerability to genotoxic insults, including ionizing radiation, environmental toxins, and chemotherapy. These agents induce DNA damage that ultimately impairs SSC function [4–6].

Similar to other tissue-derived adult stem cells, SSCs are highly sensitive to DNA double-strand breaks (DSBs), which compromise genomic integrity [7–9]. The efficient repair of DSBs is therefore critical for preventing stem cell exhaustion or the accumulation of mutations. In mammalian cells, DSBs are primarily resolved through two principal pathways: non-homologous end joining (NHEJ) or homologous recombination (HR) [10, 11]. In response to damage, SSCs activate a robust DNA Damage Repair (DDR) pathway which involves regulators such as P53, 53BP1, KU70, DNA-PKcs, MDC1 and P21 [12]. Our previous studies demonstrated that SSCs utilize a tissue-specific, γH2AX-independent repair pathway and preferentially engage the error-prone NHEJ mechanism in a manner critically dependent on 53BP1 [13, 14]. Single-cell RNA sequencing (scRNA-seq) has further indicated that reduced DNA repair efficiency as a potential mechanism underlying age-related infertility in SSCs [15]. Collectively, these findings underscore the imperative to elucidate the specific mechanisms governing NHEJ in SSCs, which could provide a foundation for novel diagnostic and therapeutic strategies for male infertility.

SIRT1 is an NAD⁺-dependent deacetylase that plays a key role in regulating DDR and maintaining genomic stability across many cell types [16, 17]. Through deacetylation of key protein targets, such as P53, KU70, and FOXO1, SIRT1 modulates multiple critical cellular processes [18, 19]. Within the male germline, the study of SIRT1-deficient mice has established a direct link between this deacetylase and fertility, as these models develop infertility due to defective spermatogenesis [20]. Although the function of SIRT1 in DNA repair pathways, particularly in its capacity as a modulator of NHEJ, has been extensively characterized in somatic cells [21], its specific role within SSCs remains poorly defined. Given the established involvement of NHEJ in the DDR pathways of SSCs, elucidating the regulatory influence of SIRT1 on the process is crucial for advancing our understanding of the molecular mechanisms underpinning male reproductive health.

In this study, we sought to define the role of SIRT1 in the DNA damage response of SSCs by integrating human single-cell transcriptomic evidence with complementary experimental models. We combined reanalysis of a published human testicular scRNA-seq dataset with clinical tissue validation and mechanistic studies in SSC systems to determine how SIRT1 influences DNA repair capacity under genotoxic stress. In particular focus on KU70, we further explored the functional relationship between SIRT1 and the NHEJ machinery to clarify how SSCs maintain genomic integrity. Our findings elucidated novel mechanisms underlying SSC genomic maintenance and suggested potential therapeutic insights for treating male infertility associated with genotoxic stress.

## Methods

### scRNA-seq reanalysis

Public single-cell RNA-seq data were downloaded from Gene Expression Omnibus (GEO, GSE149512), including three OA controls (GSM4504182, GSM4504185, GSM4504191) and three NOA samples (GSM4504195, GSM4504196, GSM4504197). Raw count matrices were processed in R using Seurat. Low-quality cells (nFeature_RNA < 200 or mitochondrial reads >20%) were removed, and doublets were excluded using DoubletFinder. Data were log-normalized, the top 2,000 variable genes were selected, and OA/NOA datasets were integrated using canonical correlation analysis (CCA) to correct batch effects. Principal component analysis (PCA) and uniform manifold approximation and projection (UMAP) were used for dimensionality reduction, and clusters were identified by graph-based clustering (resolution = 0.5). SSCs were annotated based on canonical markers (*GFRA1, UTF1, THY1, ZBTB16, ID4*). For differential expression, pseudobulk counts were aggregated per sample and analyzed using edgeR (false discovery rate [FDR] < 0.05, |log2FC|>0.5). NHEJ activity was estimated as the mean expression of 11 core NHEJ genes (*XRCC6, XRCC5, PRKDC, LIG4, XRCC4, NHEJ1, DCLRE1C, POLM, PNKP, APLF, APTX*). Gene set enrichment analysis (GSEA) was performed using fgsea with Reactome DNA repair pathways.

### Human testicular specimens

Human testicular biopsy specimens were obtained from NOA patients (*n* = 3) and OA patients (*n* = 3, used as controls). NOA samples were collected during microdissection testicular sperm extraction (micro-TESE), and only cases with focal spermatogenesis were included. Clinical characteristics are provided in Supplementary Table S1. Written informed consent was obtained from all participants, and the study was approved by the institutional ethics committee (No. 2025-LHQZYYYXLL-KY-001). Biopsy specimens were used for downstream analyses, including frozen-section immunofluorescence (IF) to assess protein expression within the SSC compartment in situ.

### Animals

Male DBA/2 mice (4 weeks old; Vital River, Beijing, China) were used for SSC isolation and in vivo irradiation. Animal experiments were approved by the Medical Ethics Committee (approval no. 2025-LHQZYYYXLL-KY-001) and conducted according to institutional guidelines. Group sizes (*n* = 3 mice per group) were estimated based on our previous SSC DNA repair study and common practice in the field [13, 14], with feasibility and ethical considerations to minimize animal use. Randomization and blinding were not performed.

### Cell isolation and culture

Mouse spermatogonial stem cells (mouse SSCs; mSSCs) were isolated from the testes of 4-week-old DBA/2 male mice. Seminiferous tubules were digested with collagenase IV (1 mg/mL) followed by 0.25% trypsin/EDTA. After overnight pre-plating on gelatin-coated dishes to remove adherent somatic cells, non-adherent cells were transferred onto mitomycin C–treated mouse embryonic fibroblasts (MEFs). Colonies were observed after 1–2 weeks and expanded. Cells were cultured in StemPro-34 medium supplemented with nutrients and growth factors, including glial cell line–derived neurotrophic factor (GDNF, 10 ng/mL), leukemia inhibitory factor (LIF, 10³ U/mL), and basic fibroblast growth factor (bFGF, 10 ng/mL). The isolation and culture protocol was adapted from our previous study on DNA repair in SSCs [13].

The C18-4 mouse spermatogonial stem cell line was obtained from BLUEFBIO™ and cultured under the vendor-recommended conditions. Cells were routinely confirmed to be mycoplasma-free.

### DSBs induction in vivo and in vitro

For in vivo experiments, mice were irradiated with 2 Gy X-rays (RX-650, Faxitron; 160 kV, 6.3 mA, 1 Gy/min) [22], and testes were collected at 30 min, 2 h, and 24 h post-irradiation. For in vitro assays, mSSCs were irradiated under the same X-ray conditions as previously described [13, 14].

For hydroxyurea (HU)-induced DNA damage, C18-4 cells were treated with HU (3 mM, 24 h), based on dose–response testing (1–50 mM, 24 h) with 53BP1 foci assessment [23]. Control groups received 0 Gy irradiation or vehicle.

### IF staining and microscopy

IF staining was performed on human testicular tissues, mouse testes, and cultured mSSCs. For tissue IF, specimens were fixed in 4% paraformaldehyde, cryoprotected in 15–30% sucrose, embedded in optimal cutting temperature compound (OCT), and sectioned at 10 μm. For cell IF, mSSCs were plated on laminin-coated coverslips, fixed at the indicated time points after DNA damage induction, permeabilized, and blocked with 0.1% Triton X-100. Samples were incubated with primary antibodies (e.g., SIRT1, KU70, 53BP1, PLZF, and AKL5C1; details in Supplementary Table S2) overnight at 4 °C, followed by Alexa Fluor–conjugated secondary antibodies (Jackson) and 4′,6-diamidino-2-phenylindole (DAPI) counterstaining. Human tissue sections and cultured cells were imaged by confocal microscopy under identical settings across groups and analyzed using FIJI/ImageJ.

Mouse testis sections were imaged and quantified using whole-slide fluorescence imaging (WSI). When under identical settings across groups (unchanged text), regions of interest (ROIs) were randomly sampled along the seminiferous tubule basement membrane (10 fields per mouse). PLZF (+) nuclei were segmented to generate single-cell masks, and the mean fluorescence intensity (MFI) of SIRT1 and KU70 was quantified in FIJI. Co-localization was assessed by Pearson’s correlation coefficient (PCC) using Coloc2 with Costes randomization. For each mouse, ≥30 PLZF (+) cells were quantified and summarized as the median per animal; mice were treated as biological replicates for statistical testing.

All immunofluorescence experiments included immunoglobulin G (IgG) isotype controls, which are provided in the Supplementary Information (Supplementary Fig. S2).

### *Sirt1* knockdown and overexpression in SSCs

For the *Sirt1* knockdown experiment, short hairpin RNAs (shRNAs) targeting *Sirt1* (target sequence: 5′-GGA GGA TGA TGA AGA GGA A-3′) and a scrambled control (5′-TTC TCC GAA CGT GTC ACG T-3′) were cloned into the pGMLV-SC5 vector (Genomeditech, Shanghai). C18-4 cells were transfected using Lipofectamine 3000 (Thermo Fisher) according to the manufacturer’s protocol. After 48 h, cells were selected with 1.5 μg/mL puromycin for 72 h to establish stable knockdown lines.

For *Sirt1* overexpression, the full-length Sirt1 cDNA was subcloned into the same pGMLV-SC5 vector (Genomeditech, Shanghai), and C18-4 cells were transfected using Lipofectamine 3000. After 48 h, cells were harvested for downstream assays. Knockdown and overexpression efficiencies were confirmed by Western blotting using the SIRT1 antibody.

### Cell growth curve

After transfection, C18-4 cells were seeded at 5 × 10³ cells/well in 24-well plates. Cells were counted on days 1, 4, and 9 to generate growth curves. For DNA damage sensitivity assays, cells were cultured in control medium or HU (3 mM) for 24 h, and proliferation was assessed at 12 h, 24 h, and 48 h.

### Flow cytometry for apoptosis and cell-cycle analysis

Apoptosis and cell-cycle distribution were analyzed by flow cytometry in C18-4 cells (WT, scramble, sh*Sirt1*, and oe*Sirt1*). For apoptosis, cells were cultured under basal conditions or treated with HU (3 mM) for 24 h, then stained with Annexin V–fluorescein isothiocyanate/propidium iodide (Annexin V-FITC/PI) using an apoptosis detection kit (Beyotime, C1062) and analyzed immediately on a flow cytometer. For cell-cycle analysis, cells were cultured for 3 days, harvested, washed with phosphate-buffered saline (PBS), fixed in 70% cold ethanol at 4 °C, and stained with propidium iodide/ribonuclease (PI/RNase) for DNA-content profiling (Cell cycle staining kit: Beyotime, C1502). Data were processed in FlowJo with debris and doublet exclusion. Apoptosis was quantified as early (Annexin V⁺/PI⁻) plus late (Annexin V⁺/PI⁺) populations. Cell-cycle phases (G0/G1, S, and G2/M) were calculated based on DNA-content histograms.

### Western blotting

Western blotting was performed using standard procedures. Briefly, SSCs were lysed in radioimmunoprecipitation assay (RIPA) buffer supplemented with protease/phosphatase inhibitors, and protein concentrations were determined by bicinchoninic acid (BCA) assay. Equal amounts of protein (20–40 μg) were separated by sodium dodecyl sulfate–polyacrylamide gel electrophoresis (SDS–PAGE) and transferred to polyvinylidene difluoride (PVDF) membranes. Membranes were blocked with 5% non-fat milk and incubated with primary antibodies overnight at 4 °C. Primary antibodies included SIRT1, KU70, p53, phospho-p53 (Ser15), acetyl-KU70 (K331), and GAPDH (details in Supplementary Table S2). After incubation with horseradish peroxidase (HRP)-conjugated secondary antibodies, signals were developed using enhanced chemiluminescence (ECL) and imaged on a chemiluminescence system. Band intensities were quantified in ImageJ (v1.53) and normalized to GAPDH.

### Co-immunoprecipitation (Co-IP)

C18-4 cells were lysed in immunoprecipitation (IP) buffer with protease inhibitors and phenylmethylsulfonyl fluoride (PMSF). Lysates (500–1000 µg) were incubated with antibodies against SIRT1 or KU70 overnight at 4 °C, followed by Protein A/G magnetic beads (Thermo Fisher). Complexes were washed, eluted, and analyzed by Western blotting with antibodies against SIRT1 and KU70. Normal IgG and input lysates (10%) served as controls.

### NHEJ reporter assay and flow cytometry

NHEJ reporter plasmids were kindly provided by Dr. Zhiyong Mao (Tongji University, Institute of Life Science) [24]. An NHEJ reporter plasmid (pNHEJ-GFP) and pDsRed-N1 internal control were co-transfected into C18-4 cells using Lonza 4D-Nucleofector (program ES-100). Prior to transfection, the reporter was linearized by I-SceI digestion. Cells were harvested 72 h later, and green fluorescent protein/red fluorescent protein (GFP/RFP) signals were analyzed by flow cytometry (BD Biosciences). Repair efficiency was quantified as GFP⁺/RFP⁺ ratios using FlowJo v7.6.1.

### Statistical analysis

Sample sizes followed our previous SSC DNA repair study and common practice [13, 14]; ≥3 biological replicates were used with three independent experiments, and no samples/animals were excluded. Statistical analyses were performed in GraphPad Prism v9.5 (GraphPad Software). For in vivo multi–time-point data, comparisons were conducted using the Kruskal–Wallis test followed by Dunn’s multiple-comparisons test. For other datasets, Student’s t-test, one-way analysis of variance (one-way ANOVA) with Dunnett’s post hoc test, or two-way analysis of variance (two-way ANOVA) with Bonferroni correction were applied according to the experimental design. Normality and variance assumptions were assessed where applicable; when assumptions for parametric tests were not met, non-parametric tests were applied as indicated in the figure legends. Data are presented as mean ± standard deviation (SD), and *p* < 0.05 was considered statistically significant.

### Ethics approval and consent to participate

All methods were performed in accordance with the relevant guidelines and regulations. The study involving human testicular specimens and mice was reviewed and approved by the Medical Ethics Committee of Shenzhen Luohu Hospital of Traditional Chinese Medicine (approval number: 2025-LHQZYYXXL-KY-001). Informed consent was obtained from all patients who provided testicular specimens. All mouse procedures were conducted in accordance with institutional guidelines for the care and use of laboratory animals.

## Results

### Single-cell and clinical validation revealed that SSCs exhibited reduced SIRT1 expression in infertile patients, with attenuated NHEJ hallmarks

**Fig. 1:**
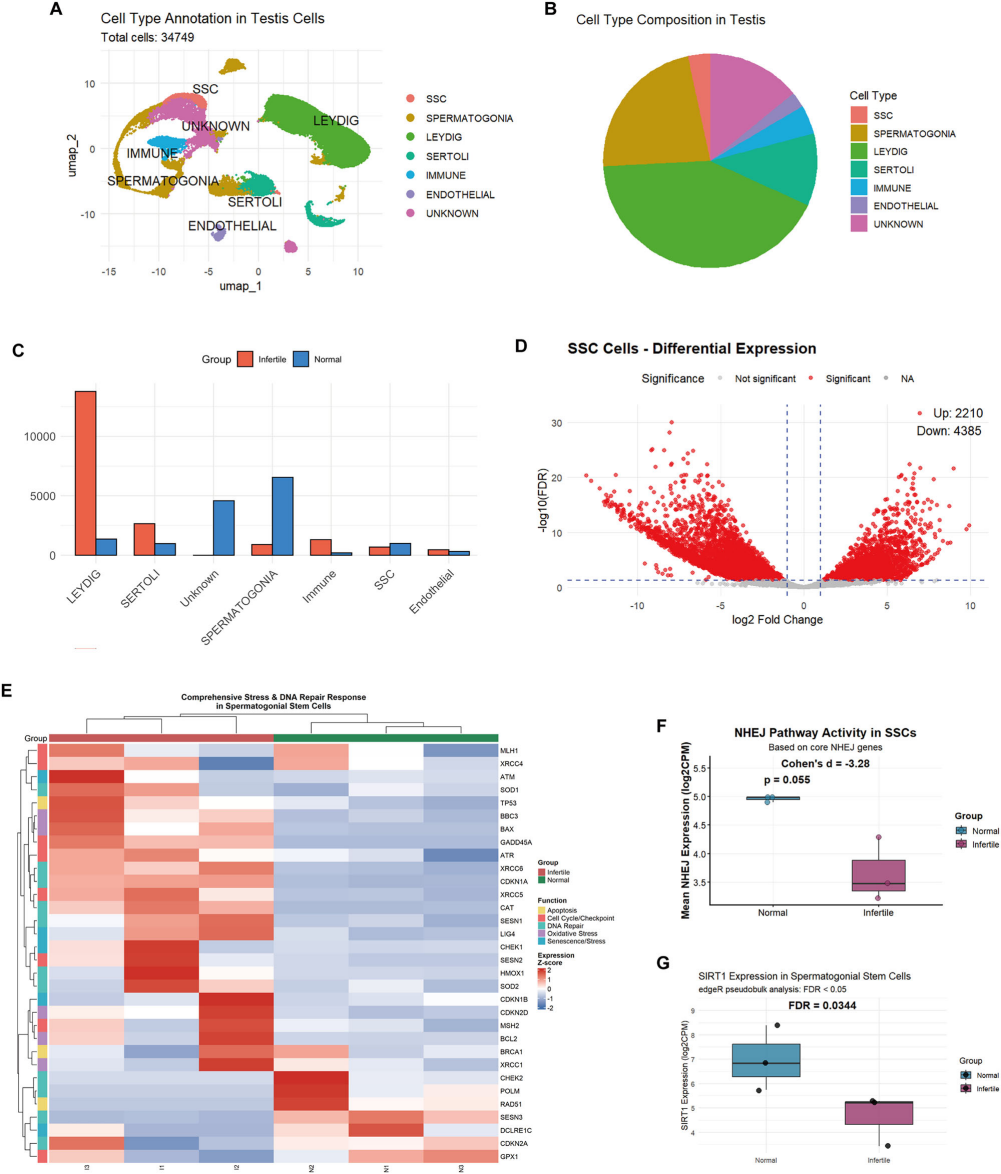
Single-cell transcriptomic analysis indicated attenuated DNA damage repair signatures and reduced SIRT1 expression in SSCs from NOA patients. **A** Cell type annotation of testicular cells. UMAP visualization of 34,749 cells identified seven major populations, including SSCs, spermatogonia, Leydig, Sertoli, immune, endothelial, and unclassified cells. **B** Cell type composition. The pie chart shows the overall distribution of cell types, with SSCs accounting for a small fraction of testicular cells. **C** Cell type distribution between groups. Quantitative comparison demonstrated a relative reduction of SSCs in NOA patients compared with controls, alongside an altered proportion of other somatic cell populations. **D** Differential expression in SSCs. Volcano plot reveals extensive transcriptional dysregulation in SSCs from NOA patients (2210 genes upregulated, 4,385 genes downregulated, FDR < 0.05, |log2FC|> 0.5). **E** Stress and DNA repair response. A heatmap of representative DNA repair and stress response genes showed widespread transcriptional abnormalities in SSCs from NOA patients. **F** NHEJ pathway activity. Mean expression of 11 core NHEJ genes showed a consistent downward trend in NOA SSCs compared with controls (Cohen’s d = –3.28, *p* = 0.055). **G** *SIRT1* expression in SSCs. Pseudobulk analysis revealed significantly decreased *SIRT1* expression in NOA SSCs (FDR = 0.0344).

To investigate the transcriptional landscape of testicular cells in NOA, we analyzed publicly available scRNA-seq datasets, including three control and three NOA samples. UMAP clustering identified seven major testicular cell populations, including SSCs, spermatogonia, Sertoli, Leydig, immune, endothelial, and unclassified cells (Fig. 1A, B). Comparison of cell type composition revealed an altered distribution of somatic cell types and a relative reduction in SSCs in NOA patients (Fig. 1C).

Differential expression analysis in SSCs demonstrated widespread transcriptional dysregulation, with thousands of genes significantly altered between NOA and control groups (Fig. 1D). Pathway-level examination highlighted abnormal expression of DNA repair and stress response genes, suggesting that SSCs from NOA patients may have impaired genome maintenance (Fig. 1E).

Focusing on the DSBs repair pathway, we observed a trend toward reduced NHEJ pathway activity, as indicated by decreased expression of 11 core NHEJ genes (Cohen’s d = –3.28, *p* = 0.055; Fig. 1F). Moreover, Pseudobulk analysis revealed that *SIRT1* expression was significantly reduced in SSCs from NOA patients (FDR = 0.0344; Fig. 1G). These results highlight NHEJ impairment and *SIRT1* downregulation as key features of NOA SSCs.

**Fig. 2:**
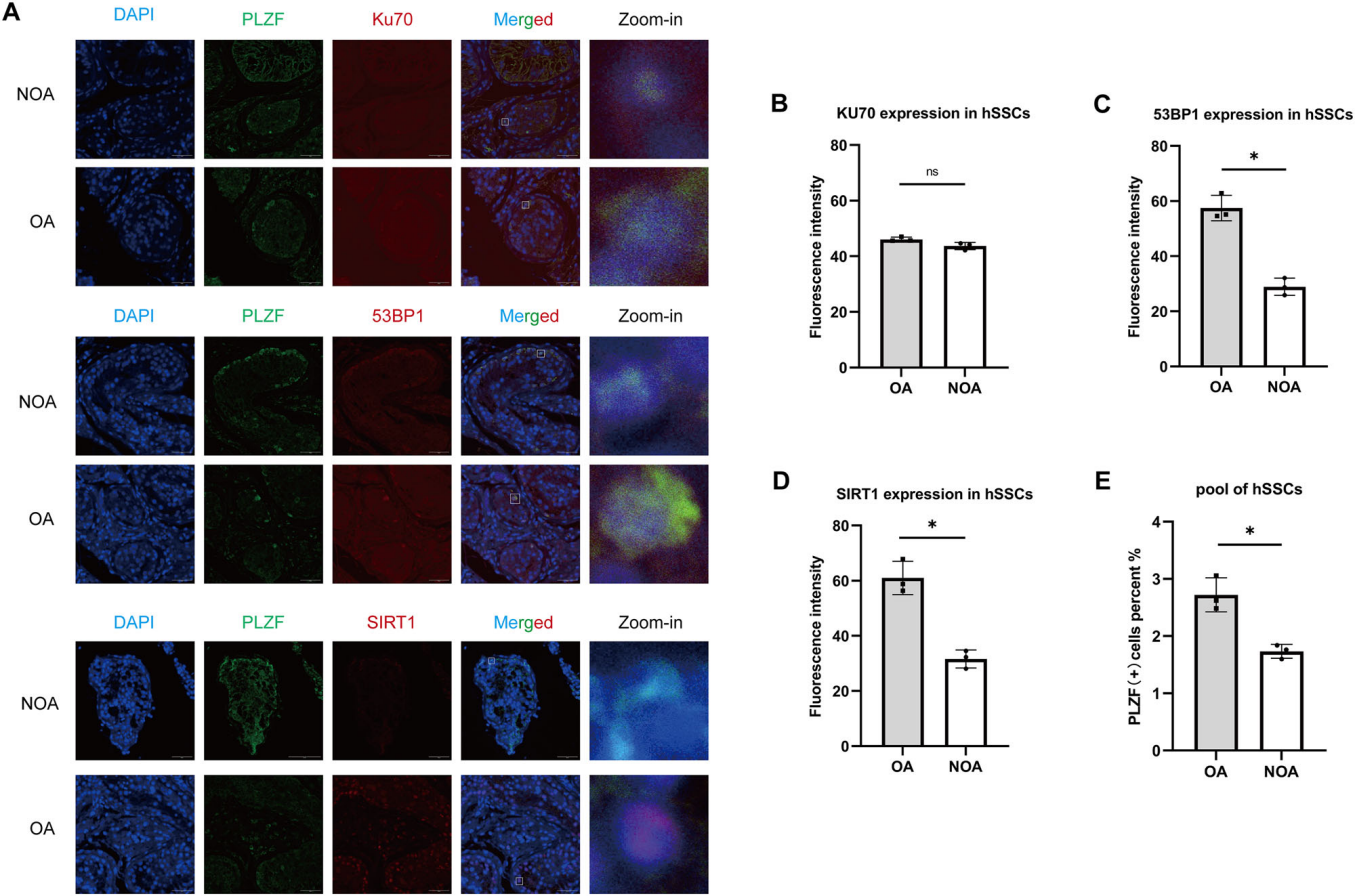
Clinical validation of PLZF (+) hSSCs and DNA damage markers in OA and NOA testicular tissue. **A** Clinical immunofluorescence validation in human testes. Representative confocal images of frozen human testicular sections from OA controls and NOA patients showing co-staining of PLZF with KU70, 53BP1, or SIRT1 (OA, *n* = 3; NOA, *n* = 3). Scale bar = 50 µm. **B**–**D** KU70, 53BP1 and SIRT1 protein expression in PLZF (+) SSCs. **E** SSC pool size. Quantification indicated a reduced PLZF ( + ) SSC pool in NOA compared with OA controls. Data are presented as mean ± SD of three biological replicates. Significance was determined by unpaired two-tailed Student’s *t* test (*p* < 0.05 was considered statistically significant; *p < 0.05; *ns,* not significant).

To further validate these findings in clinical specimens, we performed confocal immunofluorescence co-staining for PLZF and DNA damage–related markers in frozen human testicular sections from OA controls and NOA patients (OA, *n* = 3; NOA, *n* = 3) (Fig. 2A). Quantification within PLZF (+) human SSCs (hSSCs) showed that KU70 expression has no significance between two groups (Fig. 2B), whereas 53BP1 protein expression was significantly decreased in NOA hSSCs (Fig. 2C). In parallel, SIRT1 protein expression was also obviously reduced within the PLZF (+) hSSCs in NOA testes (Fig. 2D). Importantly, these changes were accompanied by a reduced hSSC pool in NOA patients (Fig. 2E). Together, the single-cell analysis and clinical validation support a reduction in hSSCs in male infertility, accompanied by downregulated SIRT1 expression and attenuated genome maintenance signatures.

### SIRT1 is dynamically involved in the SSC DDR process in vivo and in vitro

**Fig. 3:**
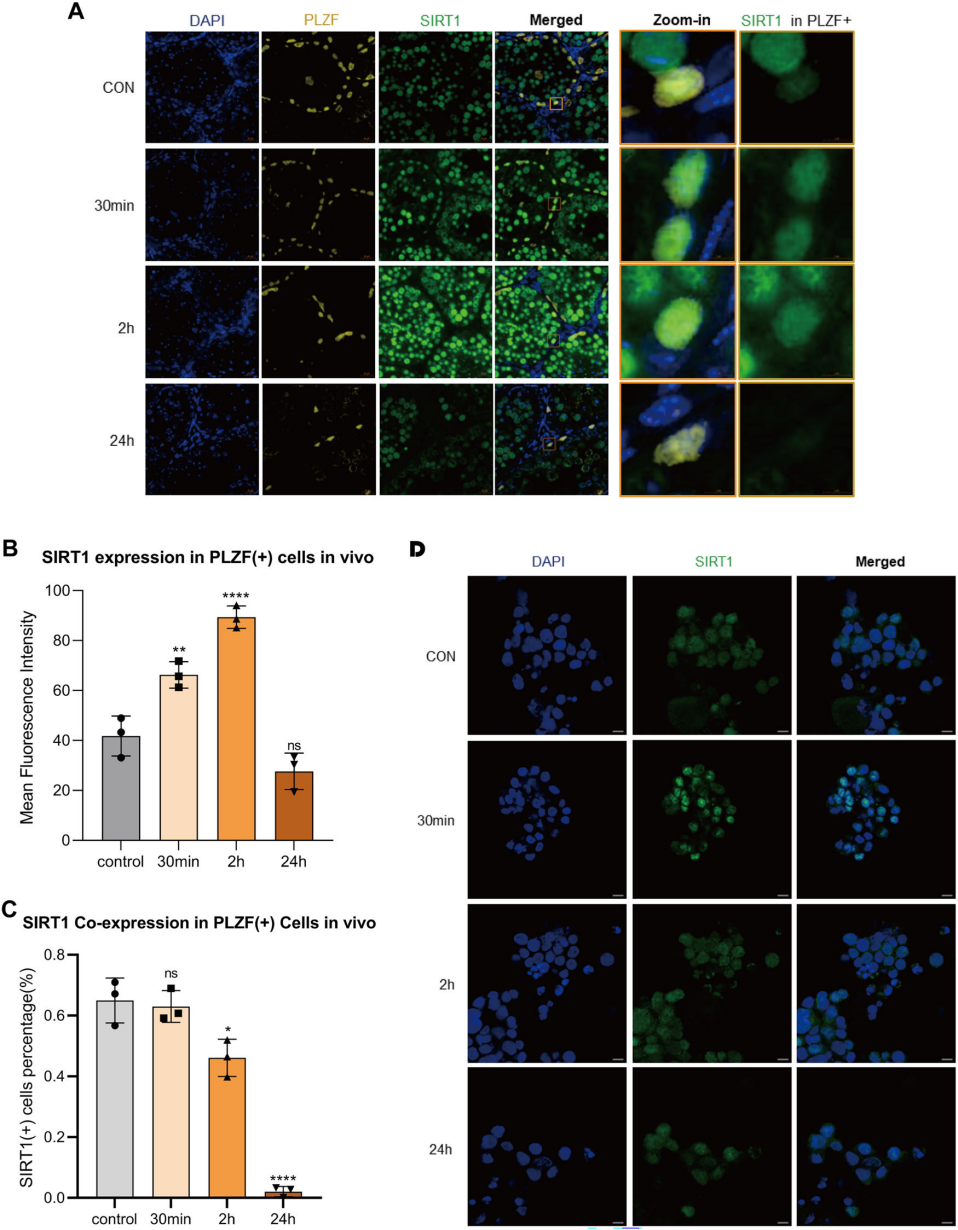
Dynamic regulation of SIRT1 in SSCs following X-ray–induced DNA damage. **A** Representative immunofluorescence images of SIRT1 (green) in PLZF (+) SSCs (yellow) from mouse testes collected at 0, 30 min, 2 h, and 24 h after 2 Gy X-ray irradiation. Nuclei were counterstained with DAPI (blue). Scale bar = 20 µm. **B** Quantification of MFI of SIRT1 in PLZF (+) SSCs at indicated time points. **C** Proportion of PLZF (+) cells co-expressing SIRT1 following X-ray irradiation. **D** Immunofluorescence staining of SIRT1 in mSSCs following in vitro exposure to 2 Gy X-ray irradiation. Scale bar, 10 µm. Data are presented as mean ± SD of three biological replicates. Significance was determined by the Kruskal–Wallis test followed by Dunn’s multiple-comparisons test (*p* < 0.05 was considered statistically significant; *ns* not significant; *p* < 0.05; *p < 0.05; **p < 0.01; ***p < 0.001; ****p < 0.0001).

To examine whether SIRT1 participates in the DDR of SSCs, DSBs were induced in vivo and in vitro. In mouse testes, IF staining showed rapid induction of SIRT1 in PLZF (+) SSCs after irradiation (Fig. 3A). Quantitative analysis demonstrated that the nuclear MFI of SIRT1 in PLZF (+) cells increased significantly at 30 min and 2 h compared with controls, then returned to baseline by 24 h (Fig. 3B). Analysis of SIRT1 co-expression within PLZF (+) cells revealed no change at 30 min, a marked decrease at 2 h, and a sharp decline at 24 h (Fig. 3C). In contrast, mSSCs irradiated in vitro exhibited a different pattern, with SIRT1 signals peaking at 30 min and progressively declining at 2 h and 24 h (Fig. 3D).

These findings indicate that SIRT1 is dynamically regulated during the SSC DNA damage response, showing sustained activation in vivo but a transient response in vitro.

### *Sirt1* knockdown/overexpression reshapes SSC proliferation and cell fate under HU-induced DNA damage

**Fig. 4:**
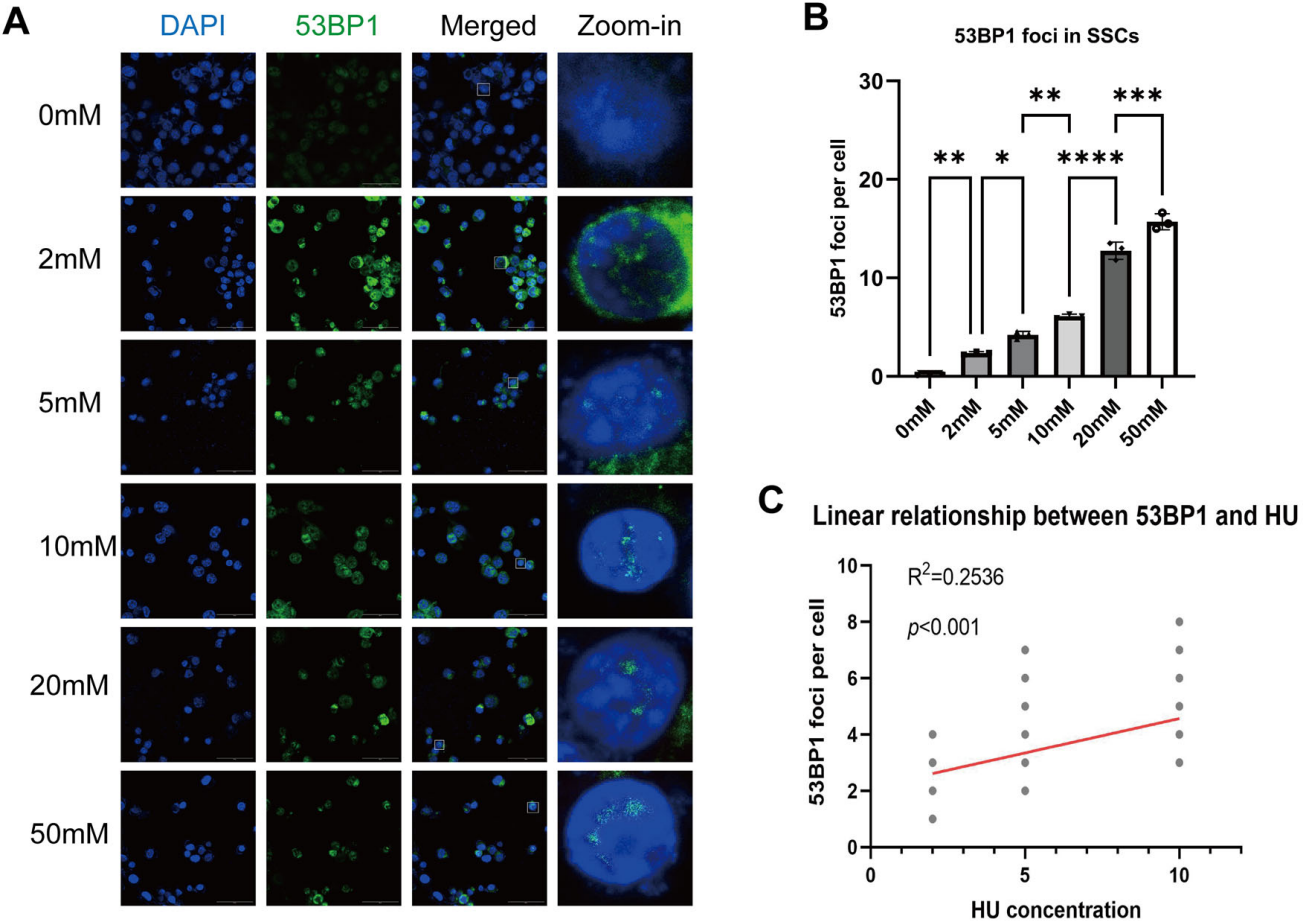
Dose-dependent induction of DNA damage signaling in C18-4 cells by HU. **A** Representative immunofluorescence images of 53BP1 foci in C18-4 cells treated with increasing concentrations of HU for 24 h. Nuclei were counterstained with DAPI. Scale bar = 50 µm. **B** Quantification of 53BP1 foci signal across HU concentrations shows a dose-dependent increase. **C** Linear regression analysis within the 2–16 mM range demonstrates a linear relationship between HU concentration and 53BP1 foci signal. Data are presented as mean ± SD of three independent experiments. Significance was determined by one-way ANOVA followed by Dunnett’s post hoc test (*p* < 0.05 was considered statistically significant; *ns*, not significant; *p* < 0.05; *p < 0.05; **p < 0.01; ***p < 0.001; ****p < 0.0001).

Prior to downstream functional assays, we first established an HU dose range that produces quantifiable DNA damage signaling in C18-4 cells. IF staining showed that HU treatment induced a progressive increase in nuclear 53BP1 foci, with increasingly prominent foci observed at higher concentrations (Fig. 4A). Consistently, quantitative analysis demonstrated a dose-dependent rise in the 53BP1 foci signal across the tested conditions (Fig. 4B). Notably, within the concentration range of 2–16 mM, the 53BP1 foci signal exhibited a linear relationship with HU concentration, supporting HU as a controllable and scalable stimulus for inducing DNA damage signaling in this cellular system (Fig. 4C). Based on these results, 3 mM HU was used in the following assays.

**Fig. 5:**
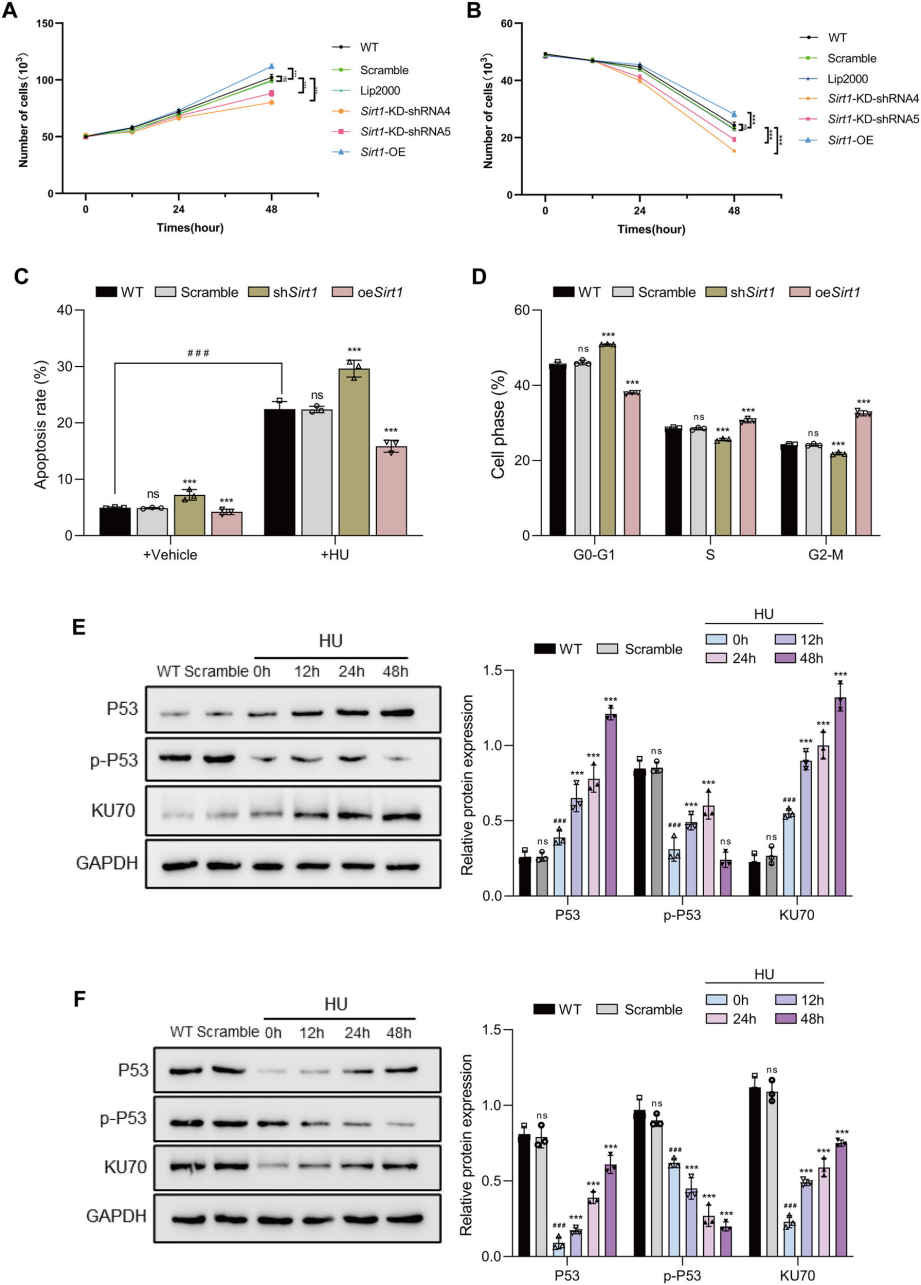
SIRT1 regulates SSC proliferation, cell-cycle progression, apoptosis, and DDR-associated protein dynamics under HU-induced DNA damage. Cell growth curves under basal conditions and HU-induced DNA damage. **A** C18-4 cells with wild-type (WT), scramble control, *Sirt1* knockdown (*Sirt1*-KD, shRNA4/5), or *Sirt1* overexpression (*Sirt1*-OE) were cultured under vehicle conditions, and cell counts were assessed at 0, 24, and 48 h. **B** Cells were treated with HU (3 mM, 24 h), and cell counts were measured at 0, 24, and 48 h. **C** Annexin V–based flow cytometric analysis of apoptosis in C18-4 cells (WT, scramble, sh*Sirt1*, and oe*Sirt1*); sh*Sirt1*(*Sirt1* knockdown); oe*Sirt1*(*Sirt1* overexpression). **D** Flow cytometric analysis of cell-cycle distribution in C18-4 cells (WT, scramble, sh*Sirt1*, and oe*Sirt1*). Western blot analysis of DDR proteins. Representative blots and quantification of P53, phosphorylated P53 (p-P53, Ser15), and KU70 expression in *Sirt1* knockdown (**E**) and *Sirt1* overexpressing (**F**), C18-4 cells were exposed to HU for 0, 12, 24, and 48 h. GAPDH was used as a loading control. Data are presented as mean ± SD of three independent experiments. Significance was determined by one-way ANOVA followed by Dunnett’s post hoc test or two-way ANOVA with Bonferroni correction (*p* < 0.05 was considered statistically significant; *ns* not significant; *p* < 0.05; *p < 0.05; **p < 0.01; ***/^# # #^*p* < 0.001; ****p < 0.0001).

To evaluate the functional role of SIRT1 in SSCs, C18-4 cells with stable *Sirt1* knockdown (KD) and overexpression (OE) were generated (Fig. S3). Under basal conditions, growth curves showed that *Sirt1* knockdown inhibited proliferation, whereas overexpression enhanced proliferation (Fig. 5A). When cells were exposed to HU-induced DNA damage (3 mM), proliferation dynamics changed in a SIRT1-dependent manner. Two-way ANOVA demonstrated that *Sirt1* knockdown enhanced HU-induced cytotoxicity and decreased cell survival compared with controls, whereas *Sirt1* overexpression reduced HU sensitivity and partially preserved cell viability (Fig. 5B). These data indicated that SIRT1 not only promotes SSC self-renewal but also modulates their susceptibility to DNA damage.

To determine whether these survival differences were accompanied by altered apoptosis, Annexin V-based flow cytometry was performed. Under vehicle conditions, neither *Sirt1* knockdown nor overexpression significantly affected apoptosis. In contrast, HU exposure markedly increased apoptosis in *Sirt1* knockdown cells, while *Sirt1* overexpression partially attenuated HU-induced cell death (Figs. 5C and S4). Together, these findings indicated that SIRT1 has a limited impact on basal apoptosis but contributes to cell survival under HU-induced DNA damage.

We next asked whether the proliferative phenotype under basal conditions was associated with altered cell-cycle progression. Cell-cycle profiling revealed a redistribution of cell-cycle phases after SIRT1 manipulation. *Sirt1* knockdown increased the fraction of cells in G0–G1 and decreased the proportions in S phase and G2–M, whereas *Sirt1* overexpression showed the opposite pattern, with more cells progressing into S phase and G2–M (Figs. 5D and S5). These data suggested that SIRT1-associated proliferative effects are, at least in part, linked to altered cell-cycle progression.

To further examine underlying mechanisms, DDR proteins were analyzed by western blotting. *Sirt1* knockdown increased KU70 and P53 levels but reduced phosphorylated P53 (p-P53, Ser15) under basal conditions. After HU treatment, KU70 and P53 were further upregulated, while p-P53 expression reappeared at later stages (12–48 h) (Fig. 5E). In contrast, *Sirt1* overexpression suppressed KU70, P53, and p-P53, with KU70 and P53 partially recovering after HU exposure, whereas p-P53 levels continued to decline (Fig. 5F). These findings suggest that SIRT1 regulates DDR protein expression, particularly KU70 and P53 signaling, during SSC DDR process.

### KU70 is dynamically involved in the SSC DDR process in vivo and in vitro

**Fig. 6:**
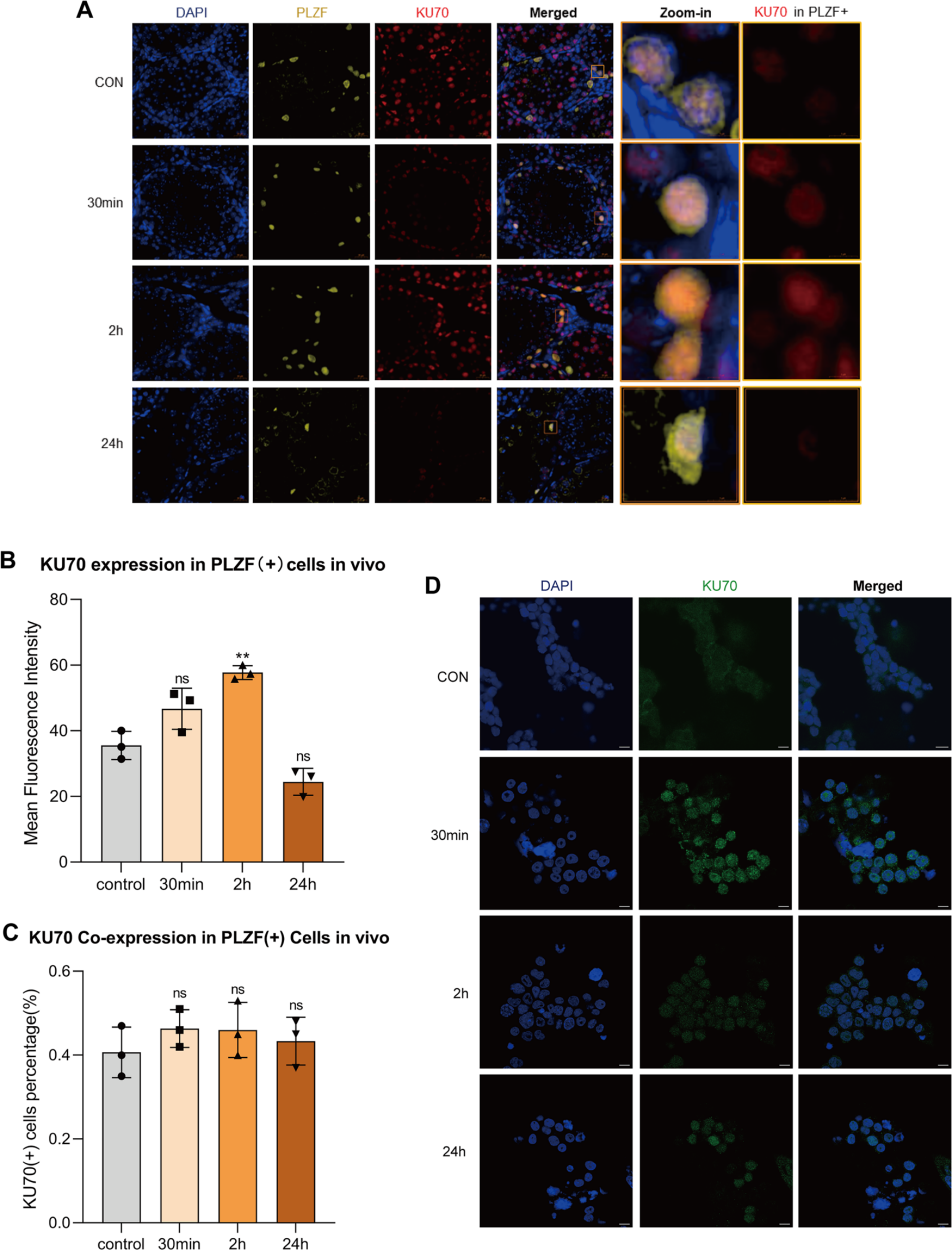
KU70 is dynamically activated during SSC DDR process. **A** Representative immunofluorescence images of KU70 (red) in PLZF (+) SSCs (yellow) from mouse testes collected at 0, 30 min, 2 h, and 24 h after 2 Gy X-ray irradiation. Nuclei were counterstained with DAPI (blue). Scale bar = 20 µm. **B** Quantification of MFI of KU70 in PLZF (+) SSCs at the indicated time points. **C** Proportion of PLZF (+) cells co-expressing KU70 following X-ray irradiation. **D** Immunofluorescence staining of KU70 in mSSCs following in vitro exposure to 2 Gy X-ray irradiation. Scale bar = 10 µm. Data are presented as mean ± SD of three biological replicates. Significance was determined by the Kruskal–Wallis test followed by Dunn’s multiple-comparisons test (*p* < 0.05 was considered statistically significant; *ns*, not significant; *p* < 0.05; *p < 0.05; **p < 0.01; ***p < 0.001; ****p < 0.0001).

Given the central role of KU70 in NHEJ, its expression dynamics in SSCs after DNA damage were examined. IF staining of mouse testis sections showed that KU70 expression in PLZF (+) SSCs was rapidly induced following X-ray irradiation in vivo (Fig. 6A). Quantitative analysis revealed that nuclear MFI of KU70 increased at 30 min and 2 h, but returned to a baseline level by 24 h (Fig. 6B). In contrast, analysis of KU70 co-expression within PLZF (+) cells showed no significant differences between irradiated and control groups (Fig. 6C), indicating that although KU70 intensity was elevated, the proportion of SSCs engaging KU70 remained relatively stable.

mSSCs exposed to X-ray irradiation in vitro exhibited a temporal pattern similar to SIRT1, with KU70 signals peaking at 30 min and gradually declining over 2–24 h in vitro (Fig. 6D).

These results demonstrate that KU70 is dynamically expressed during SSC DDR. Its nuclear intensity increases markedly after irradiation, but because most SSCs consistently express KU70, the proportion of KU70-positive cells does not change, indicating that KU70 serves as a stable repair factor broadly utilized across the SSCs population.

### SIRT1 and KU70 co-localize in SSC DNA repair

**Fig. 7:**
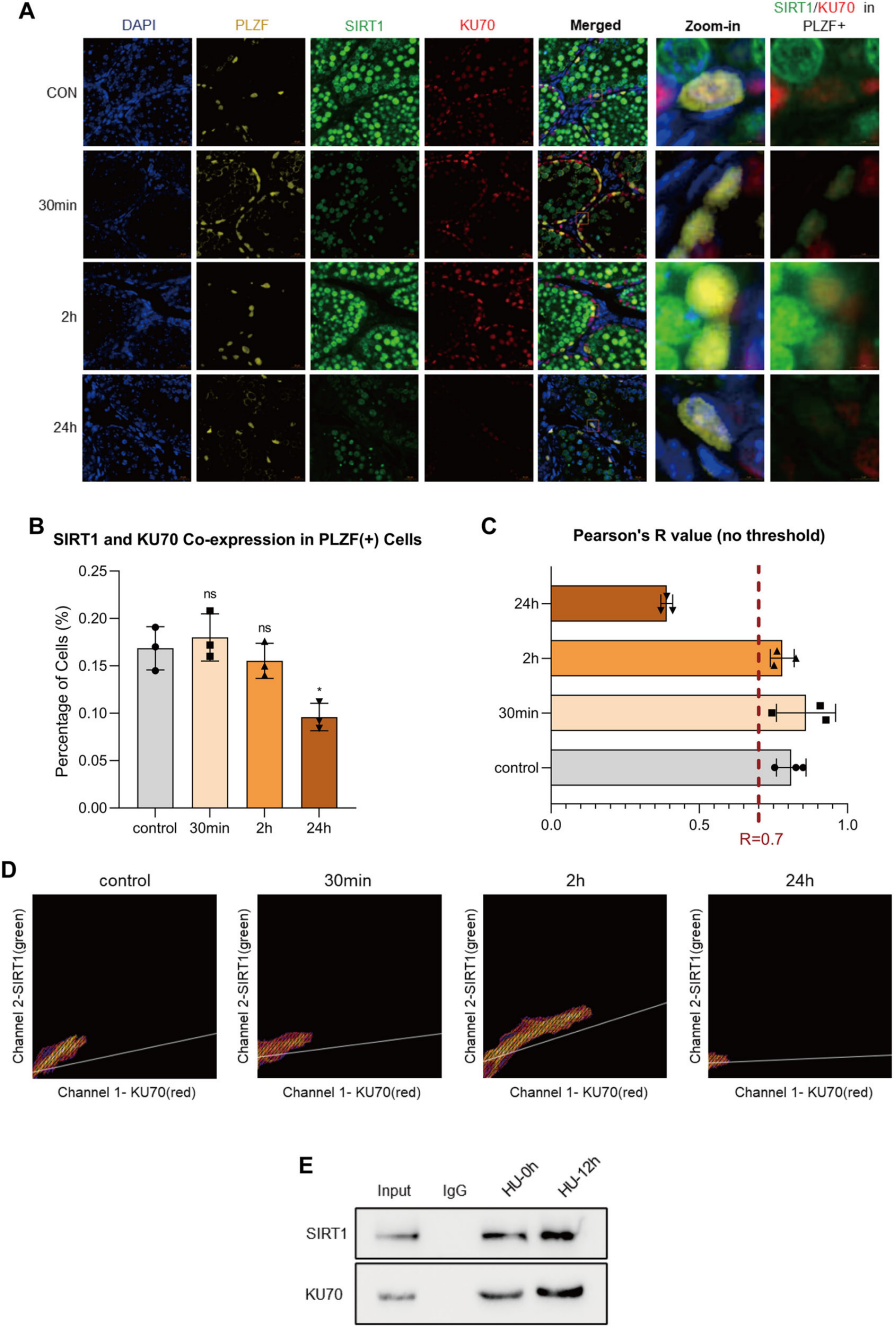
SIRT1 and KU70 co-localize and physically interact during SSC DNA repair. **A** Representative immunofluorescence images of mouse testis sections showing nuclear co-localization of SIRT1 (green) and KU70 (red) within PLZF (+) SSCs (yellow) at 0, 30 min, 2 h, and 24 h following X-ray irradiation (2 Gy). Nuclei were counterstained with DAPI (blue). Scale bar = 20 µm. **B** Quantification of the fraction of PLZF (+) cells co-expressing SIRT1 and KU70. **C** Pearson correlation coefficient (PCC) values for nuclear SIRT1 and KU70 signals at control, 30 min, 2 h, and 24 h after irradiation, with PCC thresholds (> 0.7 and < 0.7) shown for reference. **D** Representative two-dimensional intensity histograms of SIRT1 and KU70 nuclear signals at the indicated time points. **E** Co-immunoprecipitation (Co-IP) analysis of the SIRT1–KU70 interaction in C18-4 cells overexpressing *Sirt1* with or without HU (3 mM, 12 h) treatment. Data are presented as mean ± SD of three biological replicates. Significance was determined by the Kruskal–Wallis test followed by Dunn’s multiple-comparisons test (*p* < 0.05 was considered statistically significant; *ns*, not significant; *p* < 0.05; *p < 0.05; **p < 0.01; ***p < 0.001; ****p < 0.0001).

To evaluate potential colocalization of SIRT1 and KU70 during DNA repair, immunofluorescence co-staining was performed on mouse testis sections. X-ray irradiation induced clear nuclear co-localization of SIRT1 and KU70 within PLZF (+) SSCs (Fig. 7A). Quantitative analysis showed that the proportion of double-positive cells was unchanged at 30 min and 2 h compared with controls but decreased significantly at 24 h, suggesting a transient pattern of co-expression (Fig. 7B).

Colocalization analysis confirmed these dynamics. The PCC value was >0.7 in the control, 30 min, and 2 h groups, indicating a strong colocalization of SIRT1 and KU70. but declined to below 0.7 at 24 h (Fig. 7C). Two-dimensional intensity histograms further validated these results by showing tight co-distribution of SIRT1 and KU70 signals at early stages and reduced overlap at 24 h (Fig. 7D).

To corroborate these findings at the protein level, a Co-IP experiment was performed in *Sirt1*-overexpressing C18-4 cells. KU70 was successfully pulled down by anti-SIRT1 antibody, and reciprocally, SIRT1 was detected in KU70 immunoprecipitants. The interaction between SIRT1 and KU70 was markedly enhanced after HU treatment (12 h) compared with untreated controls (Fig. 7E).

These results demonstrate that SIRT1 and KU70 co-localize within SSC nuclei during DNA repair and physically interact, with their interaction strengthened under DNA damage stress.

### SIRT1 modulated NHEJ repair efficacy in SSCs

**Fig. 8:**
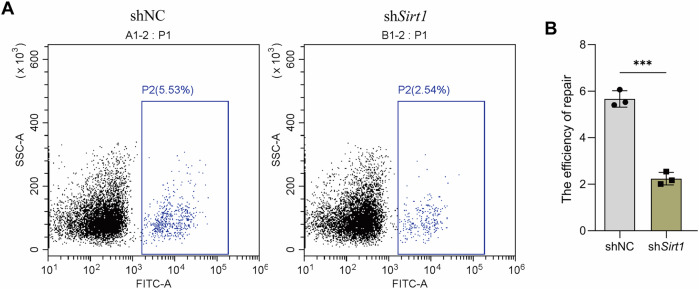
SIRT1 regulates NHEJ repair efficiency in SSCs. **A** Representative flow cytometry plot of SSCs transfected with linearized GFP-based NHEJ reporter and DsRed control plasmids under conditions of *Sirt1* knockdown (sh*Sirt1*), or controls (shNC). **B** Quantification of GFP⁺/RFP⁺ ratios as a readout of NHEJ repair efficiency after *Sirt1* knockdown (sh*Sirt1*). Data are presented as mean ± SD of three independent experiments. Significance was determined by an unpaired two-tailed Student’s *t* test (*p* < 0.05 was considered statistically significant; ***p < 0.001).

To assess whether SIRT1 regulates DSBs repair through the NHEJ pathway, a GFP-based NHEJ reporter assay was performed in C18-4 cells. After transfection with linearized reporter plasmids, flow cytometry showed that *Sirt1* knockdown significantly reduced GFP⁺/RFP⁺ ratios, indicating impaired NHEJ repair efficiency (Fig. 8A, B). These findings demonstrate that SIRT1 is required for efficient NHEJ repair in SSCs and functions as a critical regulator of genomic stability during the DNA repair process.

### *Sirt1* overexpression reduced KU70 acetylation levels in SSCs

**Fig. 9:**
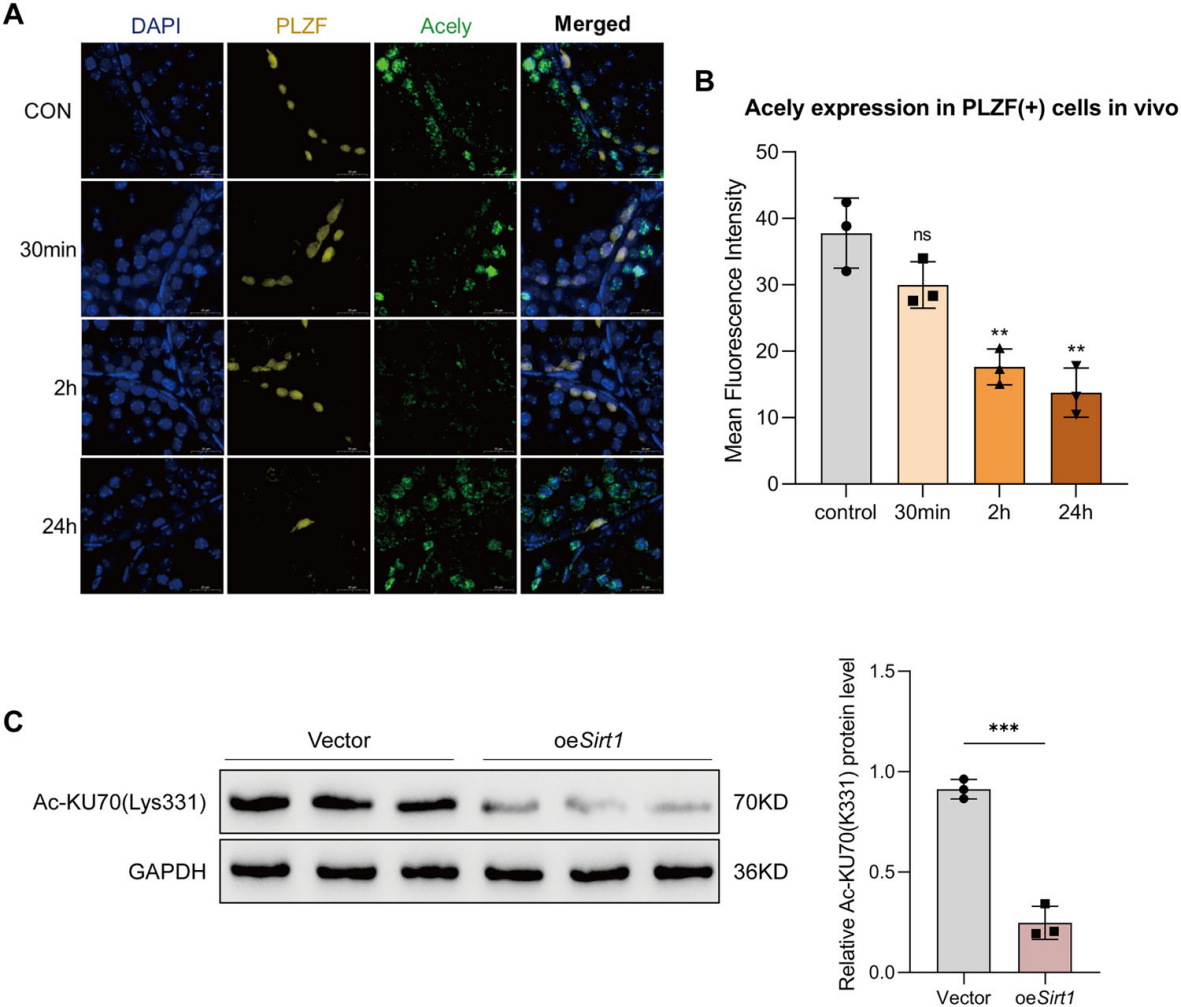
SIRT1 regulates acetylation levels in SSCs. **A** Representative immunofluorescence staining of acetyl-lysine (Acely, green) and PLZF (yellow) in mouse testis sections at baseline (control) and at 30 min, 2 h, and 24 h following X-ray irradiation. Nuclei were counterstained with DAPI (blue). Scale bar = 20 µm. **B** Quantification of MFI of acetyl-lysine in PLZF (+) SSCs at the indicated time points. **C** Western blot analysis of KU70 acetylation at lysine 331 (Ac-K331) in C18-4 cells with *Sirt1* overexpression (oe*Sirt1*) or vector control. Data are presented as mean ± SD of three biological replicates or three independent experiments. Significance was determined by the Kruskal–Wallis test followed by Dunn’s multiple-comparisons test or unpaired two-tailed Student’s *t* test (*p* < 0.05 was considered statistically significant; *ns*, not significant; *p* < 0.05; *p < 0.05; **p < 0.01; ***p < 0.001; ****p < 0.0001).

To investigate whether post-translational modifications contribute to the regulation of NHEJ in SSCs, acetylation dynamics were examined under basal and perturbed conditions. In vivo, immunofluorescence staining showed that acetyl-lysine signals within PLZF (+) SSCs progressively declined after X-ray irradiation. Quantitative analysis revealed a modest reduction of MFI at 30 min and a significant decrease at 2 h and 24 h compared with control (Fig. 9A, B).

To directly assess whether SIRT1 regulates KU70 acetylation, KU70 acetylation at lysine 331 was analyzed in C18-4 cells with *Sirt1* overexpression. Western blotting demonstrated that acetylation levels of KU70 were markedly reduced in *Sirt1* overexpressing cells compared with controls (Fig. 9C).

These findings indicate that acetylation is dynamically regulated during SSC DDR and that SIRT1 downregulates KU70 acetylation, suggesting a potential additional mechanism by which SIRT1 modulates NHEJ activity in SSCs.

## Discussion

SIRT1 participates in multiple cellular processes related to genome stability, metabolism, and reproduction by deacetylating substrates such as P53, KU70, and FOXO1[17]. Its function in DNA repair is well established in somatic cells, where it serves as a key regulator of the NHEJ pathway [21, 25]. Although *Sirt1* deficiency in mice leads to defective spermatogenesis and infertility [20], its role in SSC DDR still remains undefined. This question is particularly relevant since SSCs depend primarily on NHEJ for DSBs repair, raising the possibility that SIRT1 is a critical regulator of their genomic stability.

In the present study, from scRNA-seq analysis of human testicular samples [26], SSC transcriptional states in infertile patients were closely linked to stress responses and genome maintenance programs, consistent with genomic instability as a hallmark of NOA [14]. NHEJ-related genes tended to be downregulated in NOA SSCs relative to controls, supporting the possibility that impaired NHEJ contributes to defective spermatogenesis. SIRT1 expression was also reduced at the transcript level in NOA SSCs, and this decrease was further validated at the protein level in clinical specimens. Together, these findings suggested that SSC reduction in infertility may reflect not only impaired proliferation and differentiation, but also a reduced capacity to preserve genomic integrity under endogenous replication stress and external genotoxic insults, since unresolved DSBs can induce genomic instability and apoptosis [9].

In terms of induction of DSBs models, a previous study demonstrated that ionizing radiation doses ranging from 0.1 to 4 Gy effectively induced rational DNA damage foci [22]. Moreover, in our published work showed that 2 Gy successfully induced DSBs models both in vivo and in vitro [13, 14]; thus, 2 Gy irradiation was applied in our study. Since there are limited data on HU induction in SSCs, we referred to HU application levels in other tissue-derived stem cells [23]. We evaluated DNA damage signals across a range of HU concentrations and found that HU induced DSBs-associated 53BP1 foci. Moreover, 53BP1 foci increased approximately linearly with HU concentration between 2 and 16 mM. Therefore, 3 mM HU was applied in the present study.

Against this experimental framework, our data also highlighted an important distinction between infertility and acute genotoxic stress. Acute DNA damage may trigger a rapid stress response and transiently increase protective factors such as SIRT1 [17]. In contrast, NOA is more likely a long-term pathological condition in which niche alterations and progressive SSC loss are accompanied by reduced SIRT1 expression and weakened genome maintenance programs in the remaining SSC population [27–29].

Building on the clinical observations and the established DNA damage models, we then examined SIRT1 as a potential regulator of NHEJ in SSCs. Our results demonstrated that SIRT1 was recruited to DNA damage sites and regulated SSC homeostasis by modulating proliferative capacity under basal conditions and influencing apoptosis and cell survival under HU-induced DNA damage. Notably, our cell-cycle profiling provides additional support that the proliferative phenotype reflects altered cell-cycle progression rather than cell loss. The role of SIRT1 influence on proliferation varies across biological systems [30, 31]. In many differentiated somatic or cancer contexts, SIRT1 activity has been linked to proliferation restraint [32], whereas in other settings it supports growth [33]. Such variability likely reflects differences in cell identity, basal stress burden, NAD availability, and the dominant set of SIRT1 substrates engaged [31, 34]. In stem cells, sustained proliferation is tightly coupled to genome integrity, because even modest endogenous damage can activate checkpoints and slow cell-cycle progression [35–37]. Under this framework, SIRT1 may support proliferation capacity indirectly by limiting damage accumulation and reducing persistent checkpoint signaling. This hypothesis is consistent with our findings in SSCs, where changes in proliferation occurred without a concomitant change in basal apoptosis, suggesting that the growth phenotype cannot be simply attributed to cell death.

Our functional results also found that SIRT1 modulates the expression of KU70 and P53/p-P53, which are key mediators of DDR pathways [38]. Since KU70 is a central component of NHEJ [39], we further analyzed its role alongside SIRT1. We found that both factors contributed to SSC DDR, but their activation patterns diverged depending on context. In the testicular niche, SIRT1 and KU70 were persistently upregulated after X-ray–induced DSBs, indicating that SSCs in their niche environment maintain prolonged DNA repair activity. In contrast, mSSCs only showed short-term activation in vitro, with an early peak that declined rapidly, suggesting that essential microenvironmental signals for sustaining repair are absent in vitro [29, 40]. These findings may indicate the critical role of the testicular microenvironment in supporting SSC repair capacity.

Mechanistically, SIRT1 was found to interact directly with KU70, and this interaction, confirmed by colocalization and co-immunoprecipitation assays, was enhanced after DNA damage. NHEJ reporter assays demonstrated that *Sirt1* knockdown reduced repair efficiency, providing direct evidence that SIRT1 regulates NHEJ through binding to KU70 and maintaining its basal expression. Post-translational regulation was also observed in SSCs; DNA damage was associated with reduced acetyl-lysine signals, and *Sirt1* overexpression further decreased KU70 acetylation, consistent with previous reports in somatic cells that SIRT1 deacetylates KU70 to modulate DNA repair [18]. These findings support a role for SIRT1-mediated KU70 deacetylation in germline stem cells, although its precise contribution to NHEJ activity requires further mechanistic validation.

Another important observation was that at the early stage of the DDR (30 min), the proportion of SIRT1–KU70 double-positive SSCs remained unchanged, indicating stable activation of this repair axis. As repair progressed, the fraction of SIRT1-positive and double-positive SSCs declined, while the proportion of KU70-positive cells remained constant. This pattern suggests that KU70 is constitutively expressed in SSCs, whereas SIRT1 is the limiting regulator determining sustained repair activity, underscoring its role as a critical regulator of SSC DNA repair and a potential therapeutic target in NOA.

In conclusion, our study suggested that SIRT1 is a stress-responsive regulator of SSC fate under genotoxic challenge. By supporting NHEJ-associated repair capacity through functional cooperation with KU70, SIRT1 may increase the ability of SSCs to withstand DNA damage and thereby help to preserve the SSC pool. In the NOA testicular, reduced SIRT1 within the SSC compartment is accompanied by weakened genome maintenance programs, a pattern consistent with greater vulnerability to unresolved DNA lesions, genomic instability, and apoptosis. These findings linked a defined DDR regulatory axis to a clinically relevant stem cell reduction phenotype and supported a disease-oriented model in which impaired DNA repair contributes to a shift from SSC maintenance toward progressive attrition. Together, this work highlighted SIRT1-related pathways as potential targets to limit genotoxic injury-driven SSC loss and to support fertility preservation.

### Limitations and future directions

This study has several limitations that should be acknowledged. First, the clinical validation cohort is necessarily small, reflecting the challenges of obtaining NOA specimens and the rarity of SSCs in human biopsies. Second, although we established a link between SIRT1 and KU70 regulation that promotes NHEJ efficiency, a direct causal relationship between KU70 acetylation and repair outcomes requires further validation through mutations at the KU70 acetylation sites. Third, our transcriptomic analyses relied on a limited number of NOA samples; expanding to larger patient cohorts and employing spatial transcriptomics would more comprehensively elucidate SSC heterogeneity and the dysregulation of DNA repair pathways. Moreover, while we demonstrated SIRT1’s role in SSCs proliferation and DNA repair, its potential cooperativity with other core NHEJ factors such as XRCC4 and LIG4 [10] still needs further exploration. Finally, long-term in vivo studies using *Sirt1* conditional knockout mouse models are necessary to definitively establish the physiological relevance of our findings for fertility outcomes.

Future work should focus on elucidating the interplay between the SSC niche signaling environment and SIRT1-mediated repair, in addition to exploring potential therapeutic strategies aimed at enhancing SIRT1 activity. These research directions could yield novel avenues to preserving SSC genomic integrity and improving clinical fertility preservation strategies for men facing gonadotoxic insults from radiation, chemotherapy, or environmental toxins.

## Supplementary information

supplementary data

original data

## Data Availability

The datasets generated and analyzed during the current study are available from the corresponding author upon reasonable request.
