# Supplementary material for: SIRT1 mediates KU70 to maintain genomic stability in spermatogonial stem cells via the NHEJ repair pathway
Source: Cell Death Dis. 2026 Apr 7;17(1):490. doi: 10.1038/s41419-026-08710-4 (PMC13187197; doi:10.1038/s41419-026-08710-4)
Supplement: Supplementary file 1 — supplementary data [file 41419_2026_8710_MOESM1_ESM.pdf]

## Table S1. Detailed clinical characteristics of enrolled patients

| Name | Age | Diagnosis | Notes |
|---|---|---|---|
| Cheng Jixun | 32yr | NOA | Sperm were retrieved by micro-TESE |
| Zhou Liqiu | 35yr | NOA | Clinical pregnancy achieved following micro-TESE |
| Ni Songliang | 28yr | NOA | Clinical pregnancy achieved following micro-TESE |
| Chen Lei | 33yr | OA | Sperm retrieval via conventional TESE |
| Ding Yi | 28yr | OA | Sperm retrieval by percutaneous sperm aspiration |
| Shi Hao | 32yr | OA | Sperm retrieval by testicular sperm aspiration |

## Figure S2. Immunofluorescence analysis of protein expression with isotype-matched IgG controls.

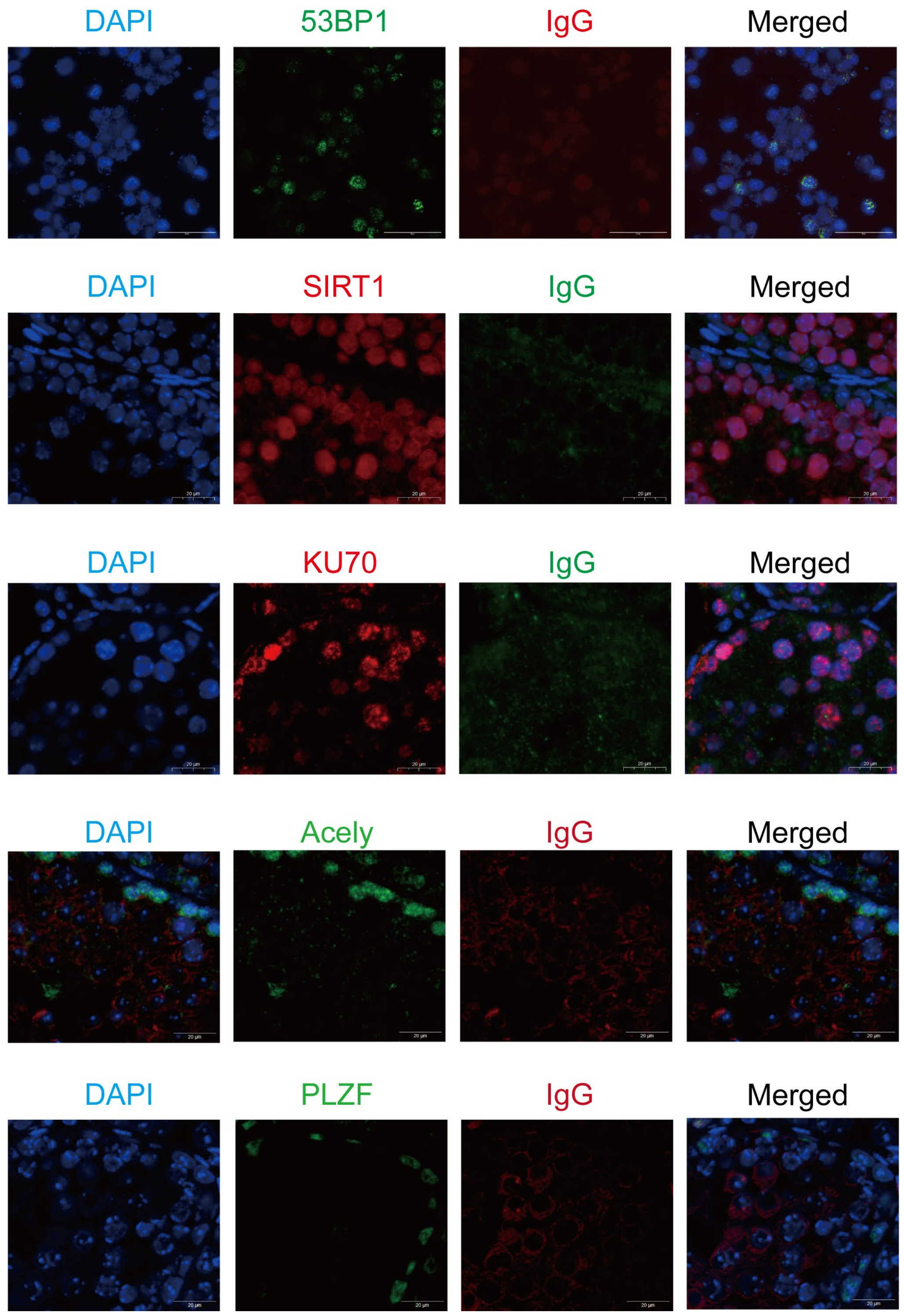

Figure S3. *Sirt1* knockdown and overexpression efficiencies were confirmed by Western blotting

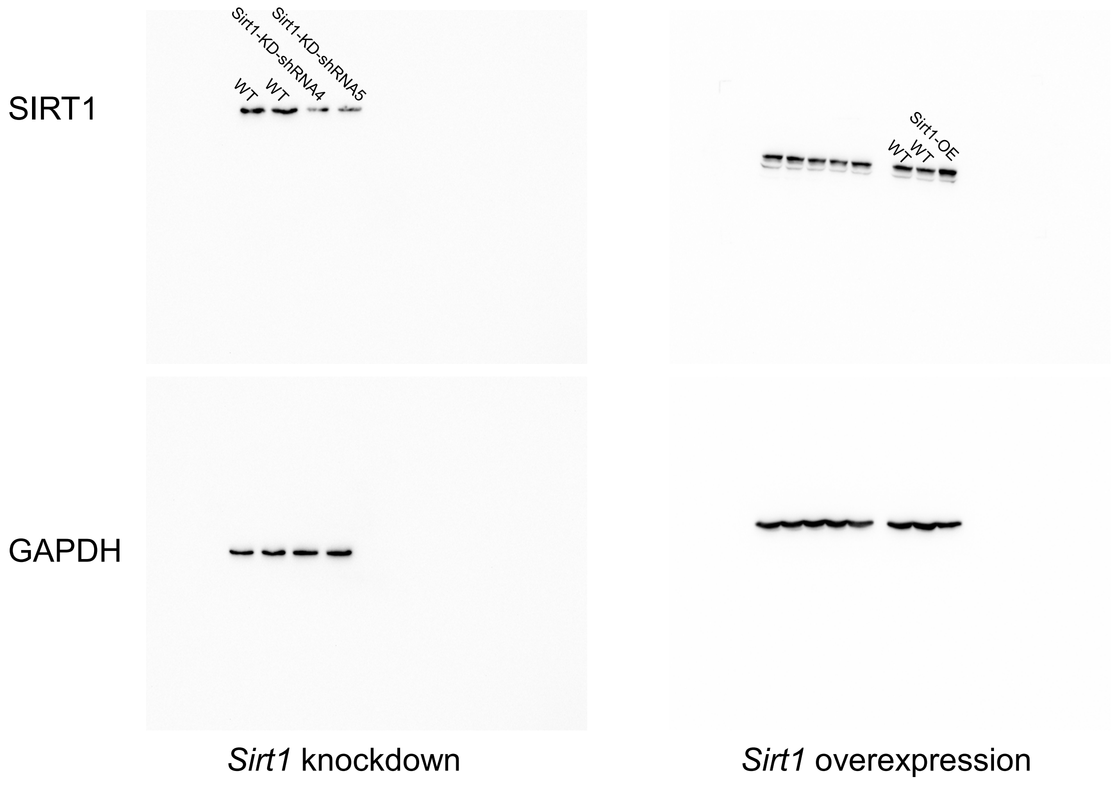


Figure S4. Flow cytometry analysis of apoptosis in C18-4 cells following *Sirt1* knockdown and overexpression.

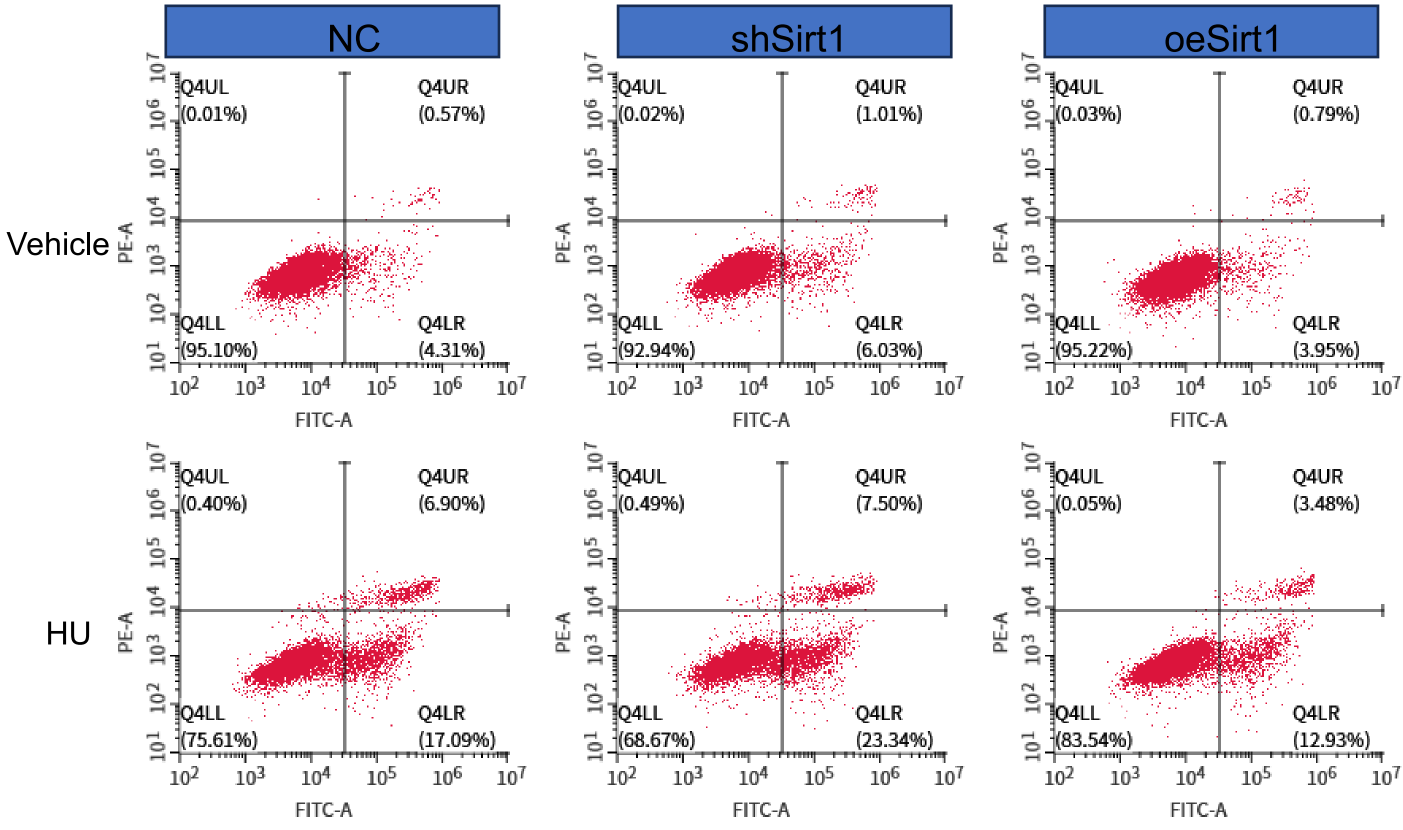

Figure S5. Flow cytometry analysis of cell-cycle in C18-4 cells following *Sirt1* knockdown and overexpression.

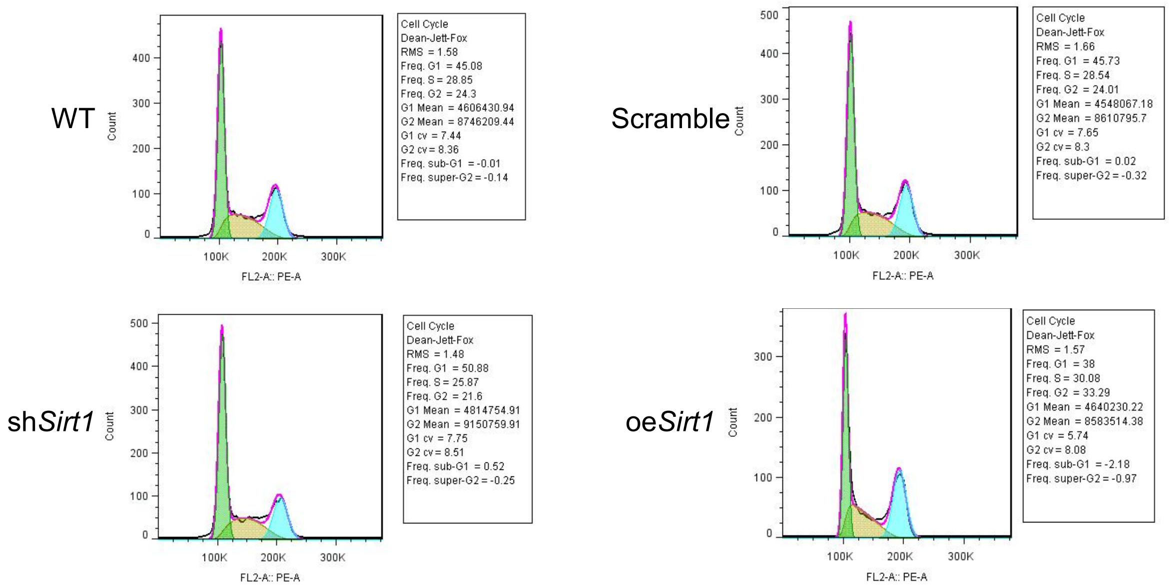


Table S2. Antibody information in present study

| **Antibody** | **Target** | **Catalog Number** | **Source** | **Dilution** |
|---|---|---|---|---|
| SIRT1 | SIRT1 | #2147331 | Millipore | 1:200 (IF), 1:1000 (WB) |
| PLZF | PLZF | sc-22839 | Santa Cruz | 1:200 (IF) |
| KU70 | KU70 | E-5 | Santa Cruz | 1:200 (IF), 1:1000 (WB) |
| 53BP1 | 53BP1 | #4937 | Cell Signaling Technology | 1:200 (IF), 1:500 (WB) |
| AKL5C1 | AKL5C1 | sc-32268 | Santa Cruz | 1:100 (IF) |
| GAPDH | GAPDH | 60004-1-Ig | Proteintech | 1:5000 (WB) |
| p53 | p53 | #2527 | Cell Signaling Technology | 1:1000 (WB) |
| phospho-p53 (Ser15) | phospho-p53 (Ser15) | ab1431 | Abcam | 1:1000 (WB) |
| KU70 (acetyl K331) | Acetylated KU70 | ab190626 | Abcam | 1:1000 (WB) |
