# Supplementary material for: SIRT1 mediates KU70 to maintain genomic stability in spermatogonial stem cells via the NHEJ repair pathway
Source: Cell Death Dis. 2026 Apr 7;17(1):490. doi: 10.1038/s41419-026-08710-4 (PMC13187197; doi:10.1038/s41419-026-08710-4)
Supplement: Supplementary file 2 — original data [file 41419_2026_8710_MOESM2_ESM.pdf]

Figure S1. Raw data of p53, p-p53, and KU70 expression in *Sirt1* knockdown

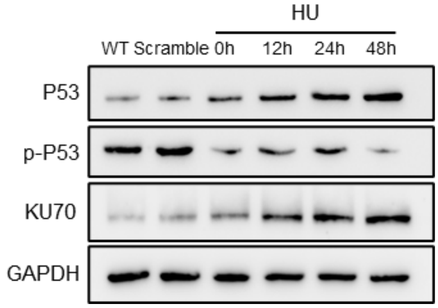


Western blot analysis of p53, p-p53, and KU70 expression in *Sirt1* knockdown



GAPDH

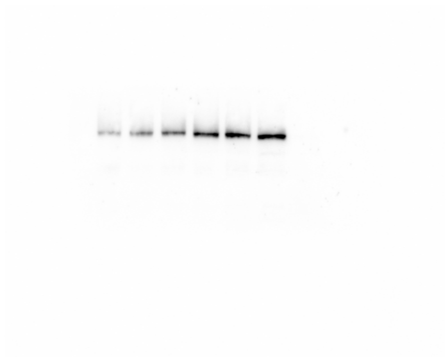

KU70



P53



p-P53

Figure S2. Raw data of p53, p-p53, and KU70 expression in *Sirt1* overexpression

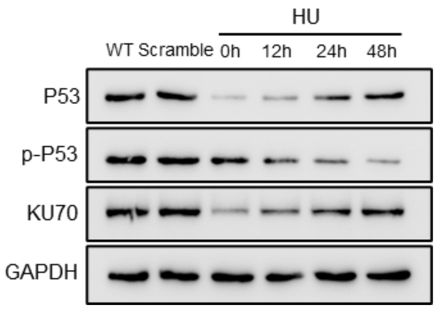


Western blot analysis of p53, p-p53, and KU70 expression in *Sirt1* overexpression



GAPDH



KU70



P53

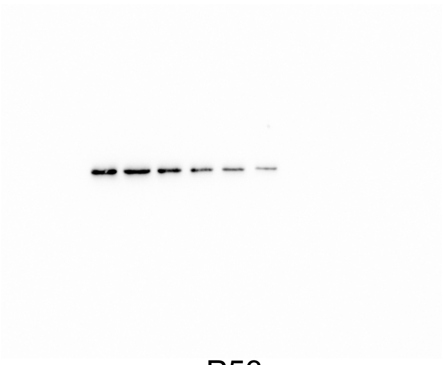

p-P53

Figure S3. Raw data of Co-immunoprecipitation

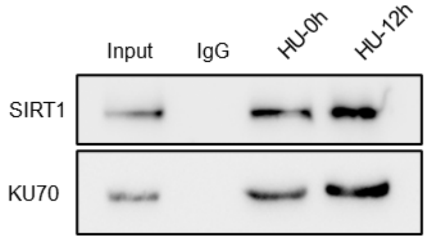






SIRT1 was detected in KU70 immunoprecipitants

KU70 was successfully pulled down by anti-SIRT1 antibody

Figure S4. Raw data of KU70 acetylation at lysine 331 in SSCs with or without *Sirt1* overexpression

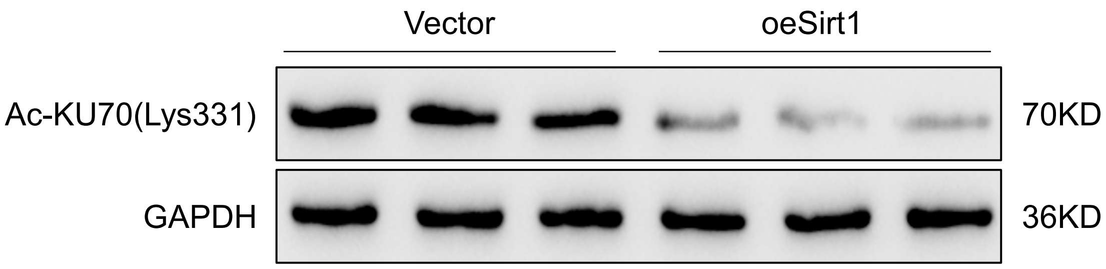






GAPDH

Ac-KU70(Lys331)

# Original data of cell curve

| TIME | GROUP | | | | average | stdev |
|---|---|---|---|---|---|---|
| 0 | WT | 49.837 | 49.278 | 48.665 | 49.260 | 0.586 |
| | Lip2000 | 48.855 | 49.976 | 48.453 | 49.095 | 0.789 |
| | Scramble | 48.419 | 48.248 | 49.780 | 48.816 | 0.840 |
| | Sirt1-KD shRNA4 | 49.732 | 49.027 | 48.901 | 49.220 | 0.448 |
| | Sirt1-KD shRNA5 | 49.913 | 48.146 | 48.108 | 48.722 | 1.031 |
| | Sirt1-OE | 48.069 | 49.737 | 48.119 | 48.642 | 0.949 |
| | | | | | | |
| 12 | WT | 46.803 | 47.104 | 46.863 | 46.923 | 0.159 |
| | Lip2000 | 46.144 | 47.243 | 47.834 | 47.074 | 0.858 |
| | Scramble | 46.410 | 47.317 | 46.820 | 46.849 | 0.454 |
| | Sirt1-KD shRNA4 | 46.333 | 47.370 | 47.269 | 46.991 | 0.572 |
| | Sirt1-KD shRNA5 | 46.985 | 46.073 | 47.691 | 46.916 | 0.811 |
| | Sirt1-OE | 47.382 | 47.462 | 46.245 | 47.030 | 0.681 |
| | | | | | | |
| 24 | WT | 44.364 | 45.731 | 44.117 | 44.737 | 0.869 |
| | Lip2000 | 43.951 | 43.485 | 43.969 | 43.802 | 0.274 |
| | Scramble | 43.290 | 44.056 | 43.511 | 43.619 | 0.394 |
| | Sirt1-KD shRNA4 | 39.068 | 40.402 | 40.100 | 39.857 | 0.699 |
| | Sirt1-KD shRNA5 | 40.279 | 41.920 | 41.119 | 41.106 | 0.821 |
| | Sirt1-OE | 45.390 | 45.870 | 45.273 | 45.511 | 0.316 |
| | | | | | | |
| 48 | WT | 25.271 | 24.496 | 23.044 | 24.270 | 1.131 |
| | Lip2000 | 23.355 | 23.539 | 22.486 | 23.127 | 0.562 |
| | Scramble | 23.585 | 22.858 | 22.689 | 23.044 | 0.476 |
| | Sirt1-KD shRNA4 | 15.051 | 15.497 | 15.356 | 15.301 | 0.228 |
| | Sirt1-KD shRNA5 | 18.554 | 19.460 | 19.956 | 19.323 | 0.711 |
| | Sirt1-OE | 28.983 | 27.116 | 28.439 | 28.179 | 0.960 |
| | | | | | | |

+HU

Original data of cell curve

| TIME | GROUP | | | | average | stdev |
|---|---|---|---|---|---|---|
| Control | WT | 49.331 | 50.221 | 50.224 | 49.925 | 0.515 |
| | Lip2000 | 51.926 | 49.587 | 49.157 | 50.223 | 1.490 |
| | Scramble | 50.798 | 50.448 | 50.320 | 50.522 | 0.247 |
| | Sirt1-KD shRNA4 | 51.421 | 51.370 | 50.622 | 51.138 | 0.447 |
| | Sirt1-KD shRNA5 | 49.414 | 49.013 | 51.079 | 49.835 | 1.096 |
| | Sirt1-OE | 50.611 | 50.056 | 51.273 | 50.647 | 0.609 |
| | | | | | | |
| 12h | WT | 57.642 | 59.405 | 56.232 | 57.760 | 1.590 |
| | Lip2000 | 54.497 | 57.309 | 57.739 | 56.515 | 1.761 |
| | Scramble | 53.782 | 54.570 | 56.002 | 54.785 | 1.125 |
| | Sirt1-KD shRNA4 | 52.840 | 55.431 | 53.401 | 53.891 | 1.363 |
| | Sirt1-KD shRNA5 | 56.007 | 54.223 | 55.279 | 55.170 | 0.897 |
| | Sirt1-OE | 57.352 | 58.230 | 59.198 | 58.260 | 0.923 |
| | | | | | | |
| 24h | WT | 73.896 | 69.992 | 71.950 | 71.946 | 1.952 |
| | Lip2000 | 71.300 | 68.016 | 69.347 | 69.554 | 1.652 |
| | Scramble | 70.149 | 71.504 | 69.256 | 70.303 | 1.132 |
| | Sirt1-KD shRNA4 | 65.111 | 67.562 | 66.453 | 66.375 | 1.227 |
| | Sirt1-KD shRNA5 | 69.266 | 68.108 | 67.466 | 68.280 | 0.912 |
| | Sirt1-OE | 73.210 | 74.161 | 72.696 | 73.356 | 0.743 |
| | | | | | | |
| 48h | WT | 102.397 | 104.943 | 99.573 | 102.304 | 2.686 |
| | Lip2000 | 97.556 | 101.418 | 99.931 | 99.635 | 1.948 |
| | Scramble | 98.921 | 97.643 | 99.592 | 98.719 | 0.990 |
| | Sirt1-KD shRNA4 | 78.895 | 80.037 | 81.773 | 80.235 | 1.449 |
| | Sirt1-KD shRNA5 | 87.773 | 85.725 | 90.986 | 88.161 | 2.652 |
| | Sirt1-OE | 110.478 | 111.040 | 114.247 | 111.922 | 2.033 |

No HU

# Original data of cell cycle

| | G1 phase | S phase | G2 phase | SUM |
|---|---|---|---|---|
| WT-3d | 45.08 | 28.85 | 24.3 | 98.23 |
| | 46.26 | 28.61 | 23.63 | 98.5 |
| | 45.05 | 28.29 | 24.45 | 97.79 |
| average | 45.46333333 | 28.58333333 | 24.12666667 | |
| stdev | 0.690096612 | 0.280950767 | 0.436615773 | |
| | | | | |
| Scramble-3d | 45.73 | 28.54 | 24.01 | 98.28 |
| | 45.74 | 28.75 | 24.54 | 99.03 |
| | 46.75 | 28.15 | 23.92 | 98.82 |
| average | 46.07333333 | 28.48 | 24.15666667 | |
| stdev | 0.586031854 | 0.304466747 | 0.335012438 | |
| Sirt1-KD-3d | 50.88 | 25.87 | 21.6 | 98.35 |
| | 50.88 | 25.7 | 21.73 | 98.31 |
| | 51.04 | 25.22 | 22.23 | 98.49 |
| average | 50.93333333 | 25.59666667 | 21.85333333 | |
| stdev | 0.092376043 | 0.337095437 | 0.332615895 | |
| | | | | |
| Sirt1-OE-3d | 38 | 30.08 | 33.29 | 101.37 |
| | 37.67 | 31.19 | 32.32 | 101.18 |
| | 38 | 30.48 | 31.99 | 100.47 |
| average | 37.89 | 30.58333333 | 32.53333333 | |
| stdev | 0.190525589 | 0.562168421 | 0.675746501 | |

# Original data of apoptosis rate

| | live cells(%) | early apoptotic cells(%) | late apoptotic cells(%) | necrotic cells(%) | | apoptotic cells |
|---|---|---|---|---|---|---|
| A：shNC + Vector | 95.1 | 4.31 | 0.57 | 0.01 | | 4.88 |
| | 94.88 | 4.11 | 1.01 | 0 | | 5.12 |
| | 94.99 | 4.42 | 0.57 | 0.02 | | 4.99 |
| average | 94.99 | 4.28 | 0.716666667 | 0.01 | | |
| stdev | 0.11 | 0.157162336 | 0.254034118 | 0.01 | | |
| B：2.shSirt1 + Vector（Sirt1-KD） | 92.94 | 6.03 | 1.01 | 0.02 | | 7.04 |
| | 91.74 | 6.75 | 1.5 | 0.01 | | 8.25 |
| | 93.56 | 5.44 | 0.98 | 0.02 | | 6.42 |
| average | 92.74666667 | 6.073333333 | 1.163333333 | 0.016666667 | | |
| stdev | 0.925274734 | 0.656074183 | 0.291947484 | 0.005773503 | | |
| C：3.shNC + Sirt1-OE（Sirt1-OE） | 95.22 | 2.95 | 0.79 | 0.03 | | 3.74 |
| | 95.27 | 3.61 | 1.08 | 0.05 | | 4.69 |
| | 95.72 | 3.75 | 0.54 | 0 | | 4.29 |
| average | 95.40333333 | 3.436666667 | 0.803333333 | 0.026666667 | | |
| stdev | 0.275378527 | 0.427239199 | 0.270246801 | 0.025166115 | | |
| D：shNC + Vector+HU | 75.61 | 17.09 | 6.9 | 0.4 | | 23.99 |
| | 78.09 | 17.39 | 4.26 | 0.26 | | 21.65 |
| | 77.87 | 16.52 | 5.15 | 0.45 | | 21.67 |
| average | 77.19 | 17 | 5.436666667 | 0.37 | | |
| stdev | 1.372734497 | 0.441927596 | 1.34314308 | 0.098488578 | | |
| E：2.shSirt1 + Vector（Sirt1-KD）+HU | 68.67 | 23.34 | 7.5 | 0.49 | | 30.84 |
| | 71.48 | 21.12 | 6.87 | 0.54 | | 27.99 |
| | 69.25 | 20.55 | 9.56 | 0.64 | | 30.11 |
| average | 69.8 | 21.67 | 7.976666667 | 0.556666667 | | |
| stdev | 1.483543056 | 1.474075982 | 1.406923357 | 0.076376262 | | |
| F：3.shNC + Sirt1-OE（Sirt1-OE）+HU | 83.54 | 12.93 | 3.48 | 0.05 | | 16.41 |
| | 85.31 | 12.6 | 2.04 | 0.05 | | 14.64 |
| | 83.37 | 13.25 | 3.32 | 0.05 | | 16.57 |
| average | 84.07333333 | 12.92666667 | 2.946666667 | 0.05 | | |
| stdev | 1.074352518 | 0.32501282 | 0.789261258 | 8.49837E-18 | | |
